# Effect of HX108-CS supplementation on exercise capacity and lactate accumulation after high-intensity exercise

**DOI:** 10.1186/1550-2783-10-21

**Published:** 2013-04-15

**Authors:** Seung-Lyul Oh, Hyukki Chang, Hee-Jae Kim, Yong-An Kim, Dong-Sik Kim, Seong-Hyun Ho, Seon-Hee Kim, Wook Song

**Affiliations:** 1 Health and Exercise Science Laboratory, Institute of Sports Science, Seoul National University, 599 Gwanang-no, Gwanak-gu, Seoul, 151-742, Korea; 2 Department of Human Movement Science, Seoul Women’s University, Seoul, Korea; 3 ViroMed Co., Ltd, Seoul, Korea; 4 Institute on Aging, Seoul National University, Seoul, Korea

**Keywords:** HX108-CS, Schisandra chinensis, Chaenomeles sinensis, Endurance capacity, Exercise capacity, Lactate accumulation

## Abstract

**Background:**

In the present study, we determined the effects of HX108-CS (mixed extract of *Schisandra chinensis* and *Chaenomeles sinensis*) supplementation on lactate accumulation and endurance capacity. Furthermore, we examined CK (creatine kinase), LDH (lactate dehydrogenase) activity to determine whether the HX108-CS affected markers of skeletal muscle injury *in vivo* and *in vitro.*

**Methods:**

Exercise capacity was measured by an exhaustive swimming test using ICR mice divided into four groups; one group received distilled water (DW) (Control group, n = 10), and the other groups received three different dosages of HX108-CS (10, 50 and 100 mg/kg, n = 10 per group) solution in water orally. Then, for the time-dependent measurements of blood lactate, CK, and LDH, Sprague–Dawley rats were divided into two groups; one received DW (Control group, n = 10), and the other group received HX108-CS (100 mg/kg, n = 10) solution in the same way as mice. Before the exercise test, the animals were given either DW or HX108-CS for 2 weeks. High-intensity treadmill exercise was performed for 30 minutes. Blood samples were collected and analyzed during and after exercise. For the *in vitro* experiment, C2C12 cells were treated with HX108-CS to examine its effect on lactate production, CK, and LDH activity.

**Results:**

Blood lactate concentration was significantly lowered immediately after treadmill exercise in HX108-CS group; however, there were no significant differences in activities of CK and LDH between HX108-CS and control during treadmill exercise and recovery phase. Furthermore, treatment with 100 mg/kg of HX108-CS led to a significant increase in the time to exhaustion in swimming test, and concurrently blood lactate concentration was significantly decreased in 50 and 100 mg/kg treated group. Moreover, our results of *in vitro* experiment showed that HX108-CS suppressed lactate production, CK, and LDH activity in a dose-dependent manner*.*

**Conclusions:**

These results suggest that supplementation with HX108-CS may enhance exercise capacity by lowering lactate accumulation. This may in part be related to an amelioration of skeletal muscle injury.

## Background

The important of lactate metabolism for endurance performance has recently been demonstrated. The effect of lactate production on acidosis has been the topic of recent researches in the field of exercise physiology. Robergs et al., have discussed the creation of H^+^ ions that occurs during glycolysis and the production of lactate in circulating level causes acidosis and in turn that increased lactate production is one of the several causes of muscle fatigue during intense exercise [1]. Also, lower blood lactate concentrations at a given workload improve endurance exercise in various populations [2,3].

In recent years, various strategies for improvement of endurance capacity have been adopted in an attempt to attenuate the muscle damage caused by exercise. *Schisandra chinensis* and *Chaenomeles sinensis* are usually known as medicinal plants with few side effects such as toxicity. *Schisandra chinensis* is a fruit of *Schisandra chinensis* Biollon of Schisandraceae. Several studies exist on the general composition of the extract of the *Schisandra* berries and pharmacological experiments have shown its anti-fatigue effects on small rodents [4]. It has also been used as a tonic for the treatment of chronic fatigue [5], for example, in Russia, it is used for anti-fatigue material [6]. On the other hand, *Chaenomeles sinensis* is a fruit of *Chaenomeles sinensis* Koehne of Rosaceae. The fruit of *Chaenomeles sinensis* is used in traditional medicine for the treatment of cough, common cold, and pain in Korea, Japan and China. It has been reported to contain phenolic acids, flavonoids, triterpenes, and fragrant compounds. Furthermore, it has also been known to have anti-oxidant [7,8] and anti-influenza effect [9].

Several previous studies have shown that the supplementation of natural substances can decrease the contribution of exercise-induced physical fatigue and improve the animal’s physiological capacities. Administration of *Schisandra* seed extract (SSE) extended the duration of swimming of mice [10-12]. These mice that have been treated over 2 to 4 weeks period, showed increased swimming capacity even after 24 to 48 hours cessation period. In addition, the race horses treated with *Schisandra chinensis* were able to complete the race at an average of 1.8 second faster and showed lower levels of lactate after exercise, indicating an improvement in the physical performance of the horses [13,14].

To the best of our knowledge, there has been no report to examine the effectiveness of *Chaenomeles* and *Schisandra* on the exercise-induced lactate production and improvement of endurance performance. Therefore, we invented HX108-CS which contains both *Schisandra chinensis* and *Chaenomeles sinensis* extracts as active ingredients for lowering lactate accumulation and for enhancing athletic capability. In the present study, we determined the effects of HX108-CS supplement on lactate accumulation during and after high-intensity treadmill exercise. Endurance capacity was also evaluated with exhaustive swimming test. Furthermore, we measured CK and LDH activities to examine whether the HX108-CS can decrease muscle injury *in vivo* and *in vitro.*

## Methods

### Animals and surgery

Male ICR mice (4 weeks old, n = 40) and Sprague–Dawley rats (6 weeks old, n = 20) were obtained from Central Lab Animal Inc. (Seoul, Korea), kept in air-conditioned room, and acclimated for at least 7 days. The mice were divided into four groups; one group received distilled water (DW) (Control group, n = 10), and the other groups received three different dosages of HX108-CS (10, 50 and 100 mg/kg, n = 10 per group) solution in water orally. Then, the rats were divided into two groups; one received DW (Control group, n = 10), and the other group received HX108-CS (100 mg/kg, n = 10) solution in the same way as mice. Before the exercise test, the animals were given either DW or HX108-CS for 2 weeks. In the rats, they were anesthetized with zoletil 50 (10 mg/kg, i.p.; Vibac Laboratories, Carros, France) for surgery. For the blood analysis, a silicone catheter was inserted into the jugular vein and fixed by a 35 mm silk thread. The exteriorized distal end of the catheter was fixed at the animal’s nape as described in a previous study [15]. Three days after surgery, the rats were kept individually in their cages until the start of the running tests for full recovery. All experimental procedures were approved in accordance with the institutional animal care and use guidelines of the Animal Experimental Center at Seoul National University.

The procedures for handling and caring for the animals were performed in accordance with the NIH Guide for the Care and Use of Laboratory Animals. The experimental protocol was approved by the Institutional Animal Care and Use Committee of Laboratory Animal Resources at Seoul National University (SNU-100705-6).

### Preparation of HX108-CS

Total water-soluble extract of the dried fruits, HX108-CS was prepared from these hardy kiwifruits. Briefly, the dried *Schisandra chinensis* and *Chaenomeles sinensis* fruits were mixed at a 2:1 ratio by weight and extracted by boiling in DW for 3 hours. The extract was filtered with Whatman filter paper (No. 2, 110 nm), and concentrated using a rotary evaporator, followed by a freeze-drying process. Powdered HX108-CS was dissolved in DW at a concentration of 200 mg/ml and stored at −80°C until use.

### Cell differentiation and sample treatment (*in vitro*)

C2C12 cell line (kindly donated by Prof. Kang, Seoul, Korea) was routinely maintained in Dulbecco’s modified Eagle’s medium (DMEM) supplemented with 15% heat inactivated fetal bovine serum (FBS), 100 IU/ml penicillin G and 100 μg/ml streptomycin in a humidified 5% CO_2_ atmosphere at 37°C. The medium was changed three times a week. The cells were harvested and reseeded when reached by 50%.

For lactate measurement, C2C12 cells were seeded on 24-well plates (2.5 x 10^4^ cells/500 μl) in DMEM medium. C2C12 cells were treated with HX108-CS (250, 500 and 1000 μg/ml) for 30 min before NaN_3_ (sodium azide) treatment. After 30 min, NaN_3_ was treated to cells and incubated for 6.5 hrs. After 7 hrs, supernatant of cells was harvested and used for lactate measurement. For LDH and CK activities measurement, C2C12 cells were differentiated for 5 days. Briefly, C2C12 cells were seeded on 6-well plates (2 x 10^5^ cells/2 ml) in DMEM medium. To induce differentiation, the medium was replaced with DMEM containing 2% horse serum prior to the cells reaching confluence. The medium was changed every day. Differentiated C2C12 cells were treated with HX108-CS (250, 500, and 1,000 μg/ml) and 2, 4-dinitrophenyl (DNP) for 1 hr. After 1 hr, supernatant of cells was harvested and used for LDH and CK measurement.

### Swim-to-exhaustion exercise test

Each of the mice had a weight attached (10% body weight) to the tail for the duration of the swim-to-exhaustion exercise. The mice were assessed to be fatigued when they failed to rise to the surface of the water to breathe within 5 sec. Swimming time was recorded as minute for each mice. Blood samples were taken by capillary glass tubes from eye venous pool of mice when they were no longer able to continue to swim. Throughout the course of this experiment, one researcher who did not know the grouping of the mice determined the duration of swimming time to exhaustion. The swimming exercise was carried out in a tank (32 × 50 × 35 cm), filled with water to 25 cm depth and maintained at a temperature of 33 ± 1°C.

### Progressive treadmill exercise protocol

Two hours before the experiment, the rats were fasted by removing chow to minimize the glucose effect from consumption just prior to the measurement. A week before the day of experiment, all animals were familiarized to the treadmill, starting with walking on the treadmill (5 m/min) and then running at 15 m/min, 0% grade for 20 min/day, 5 days/week. The day of the experiment, the rats were placed on the treadmill at 0% slope and the speed was gradually increased up to 25 m/min for 30 min.

### Measurements of lactate, LDH and CK activity

*In vitro* study, lactate production, LDH and CK activity were measured using commercial kit (Lactate assay, Bioassay systems, USA; LDH assay, Takara, Japan; CK assay, Bioassay systems, USA). For lactate measurement, the working reagent (assay buffer, enzyme, NAD, PMS, MTT) was added into plate and tapped the plate to mix. After 0 min and 20 min, the absorbance at 565 nm was read using a 96-well plate reader. For LDH assay, the reaction mixture was added into plate and incubated for 30 min. After incubation, the absorbance at 490 nm was read using a 96-well plate reader. For CK assay, the plate was incubated at 37°C for 10 min. After 10 min and 40 min, the absorbance at 340 nm was read using a 96-well plate reader.

*In vivo* study, fatigue related parameters were evaluated as described previously [16] with some modifications. Lactate concentration (Lactate pro LT-1710, ARKRAY Inc., JAPAN) was determined from whole blood samples before, during (20 and 25 min) and immediately after exercise and recovery phases (3, 6, 9, 15 and 30 min after exercise) on the treadmill. On the other hand, LDH and CK levels were measured from whole blood samples before, immediately after exercise and recovery phases (15 and 30 min after exercise). At least 30 μl of blood sample was taken from the jugular vein catheter to measure blood lactate, LDH and CK activities. Blood CK and LDH activities were assayed using commercially available kits (LDH assay, Takara, Japan; CK assay, Bioassay systems, USA).

### Statistical analysis

Data were presented as mean ± S.E.M, and differences between vales were compared with two-way repeated measure analysis of variance (ANOVA) followed by Dunnett’s multiple comparisons with the pre-exercise value as a control. Differences between groups were tested with unpaired Student’s t-test by using Origin 8.0. *P* values less than 0.05, which were calculated as one-tailed *P* values, were considered to be statistically significant.

## Results

### Effect of HX108-CS on lactate concentration, CK, and LDH activity after high-intensity treadmill exercise

**Figure 1:**
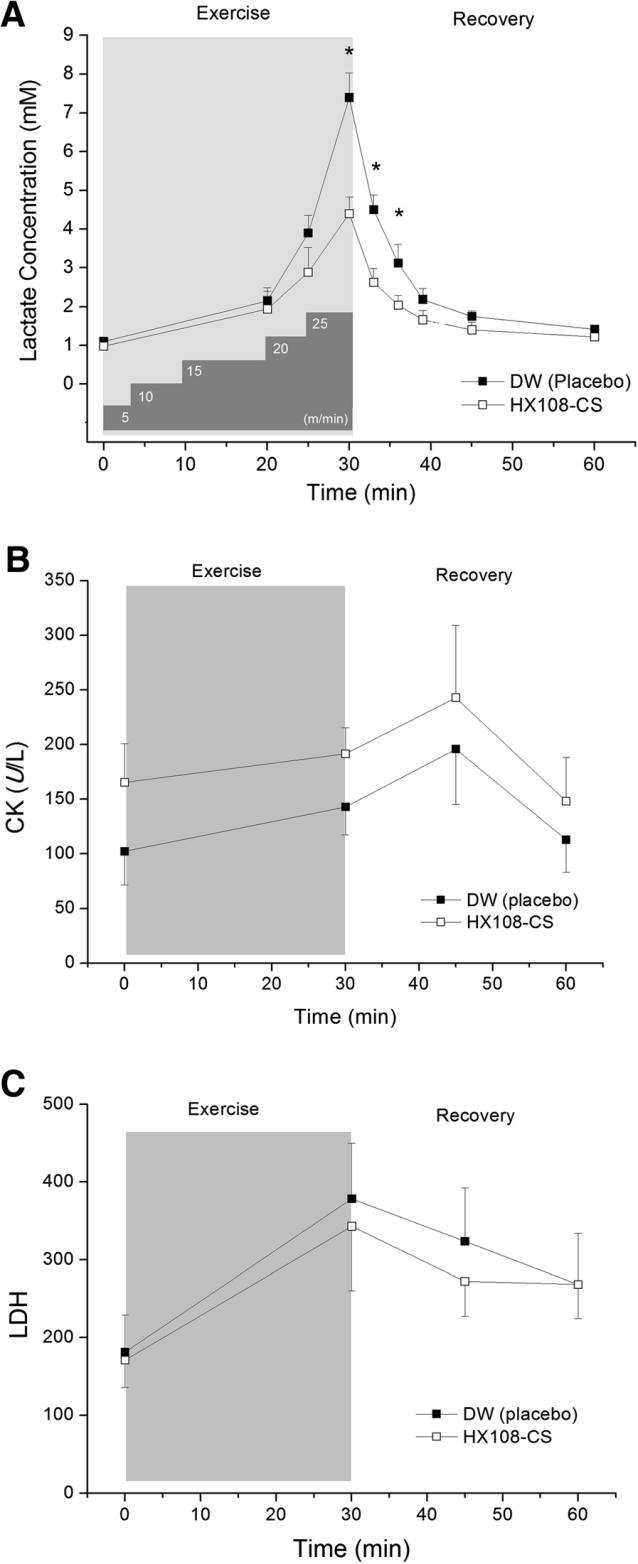
**Effect of HX108-CS on CK, LDH and lactate after progressive treadmill exercise.** Male SD rats were divided into 2 groups; DW group (DW treated, n = 7), HX108-CS treated group (100 mg/kg, n = 8). All of rats ran with the same increasing velocity from 5 m/min up to 25 m/min for 30 minutes at 0% grade (protocol was illustrated under the graph). Blood samples were collected from jugular vein before, during and after treadmill exercise. (**A**) Lactate concentration in blood, (**B**) CK activity in blood and (**C**) LDH activity in blood. Values are expressed as mean ± SEM. * p < 0.05 significant difference between DW group vs. HX108-CS treated group.

HX108-CS substantially affects both accumulation and removal of blood lactate levels during and after high-intensity treadmill exercise (Figure 1A). The lactate levels of the resting state were not statistically different between the control rats (1.1 ± 0.09 mmol/L) and HX108-CS treated rats (0.98 ± 0.10 mmol/L). However, at immediately after high-intensity exercise, the maximally elevated blood lactate concentration was significantly higher in control group compared HX108-CS group (7.4 ± 0.62 mmol/L vs. 4.4 ± 0.42 mmol/L, p < 0.05). Moreover, in early recovery phase, HX108-CS facilitated the removal of blood lactate significantly (p < 0.05) at 3 and 6 min after treadmill exercise (Figure 1A).

As shown in Figure 1B, both the control and HX108-CS groups showed a similar pattern of changes in blood concentration of CK. In both groups, CK increased immediately after exercise, and reached their maximum values at 15 min recovery phase, then returned to the pre-exercise level at 30 min recovery. On the other hand, the peak concentration of blood LDH occurred immediately after exercise in both groups then gradually decreased (Figure 1C). However, there were no significant differences in the CK and LDH between control group and HX108-CS treated group.

### Effect of HX108-CS on endurance capacity and blood lactate concentration in swim-to-exhaustion exercise test

**Figure 2:**
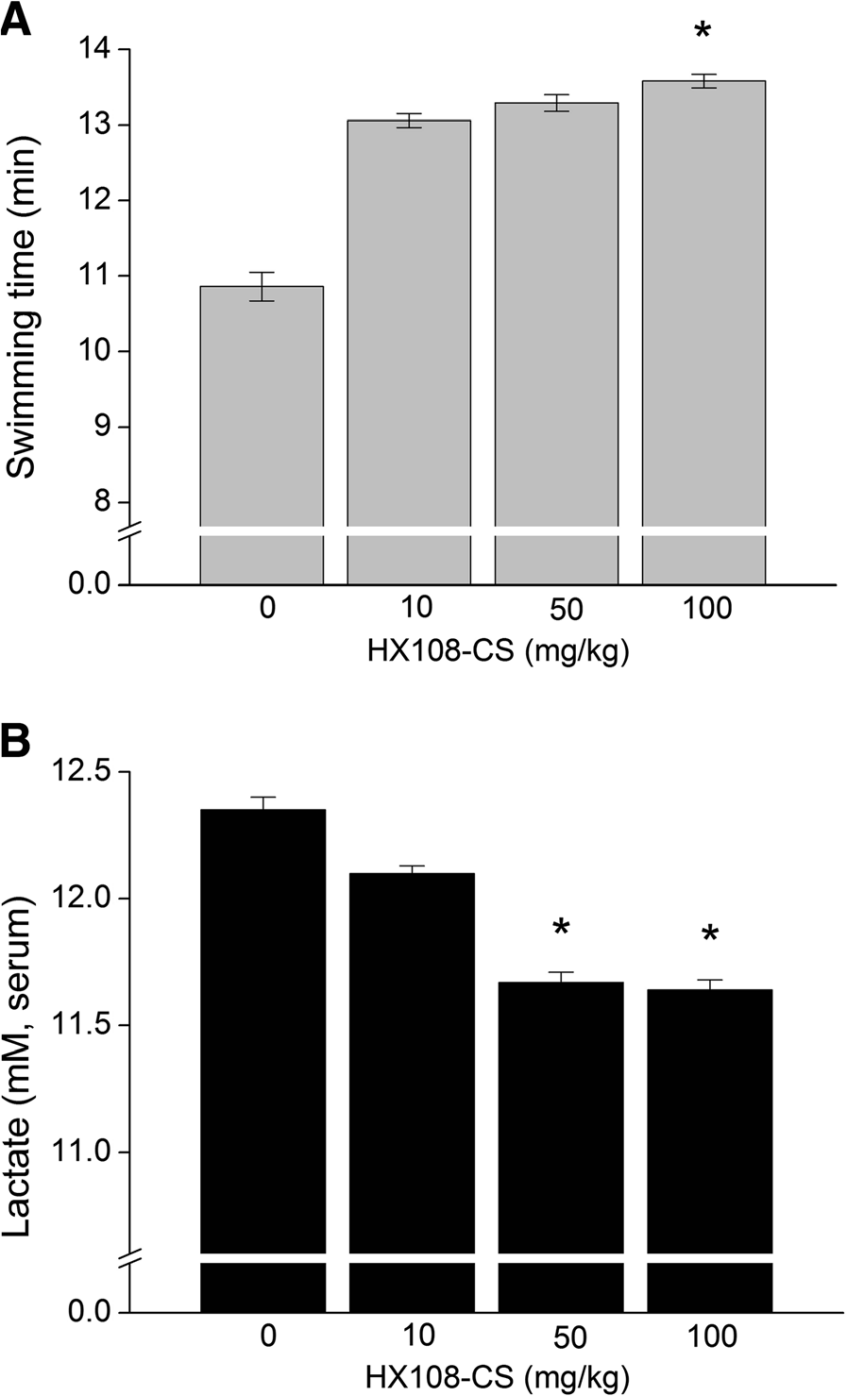
**Effect of HX108-CS on exercise capacity and lactate production in swim-to-exhaustion exercise test.** Male ICR mice were divided into 4 groups by different dosage of HX108-CS (0, 10, 50 and 100 mg/kg) and swim-to-exhaustion exercise test was performed. (**A**) Swimming time to exhaustion, (**B**) Blood lactate concentration after exhaustive swimming. Values are expressed as mean ± SEM. * p < 0.05 significant difference between control (0 mg/kg treatment) group vs. HX108-CS treated group.

We performed swim-to-exhaustion exercise test using the mice to examine the effect of HX108-CS on endurance capacity. The significant changes of endurance capacity and lactate production followed by HX108-CS supplementation were found in a dose of 100 mg/kg of HX108-CS. HX108-CS treated animals (100 mg/kg) were statistically improved the duration of swimming time to exhaustion by 24.7% compared with control group (Figure 2A, p < 0.05; swimming duration of 100 mg/kg and control were 13.58 ± 0.09 and 10.89 ± 0.19, respectively). Additionally, blood lactate concentration after exhaustive swimming was significantly decreased at 50 and 100 mg/kg of HX108-CS (p < 0.05, Figure 2B). These results indicated that HX108-CS enhanced the endurance capacity with lessening accumulation of blood lactate.

### Effect of HX108-CS on lactate, CK, and LDH activity in C2C12 cells

**Figure 3:**
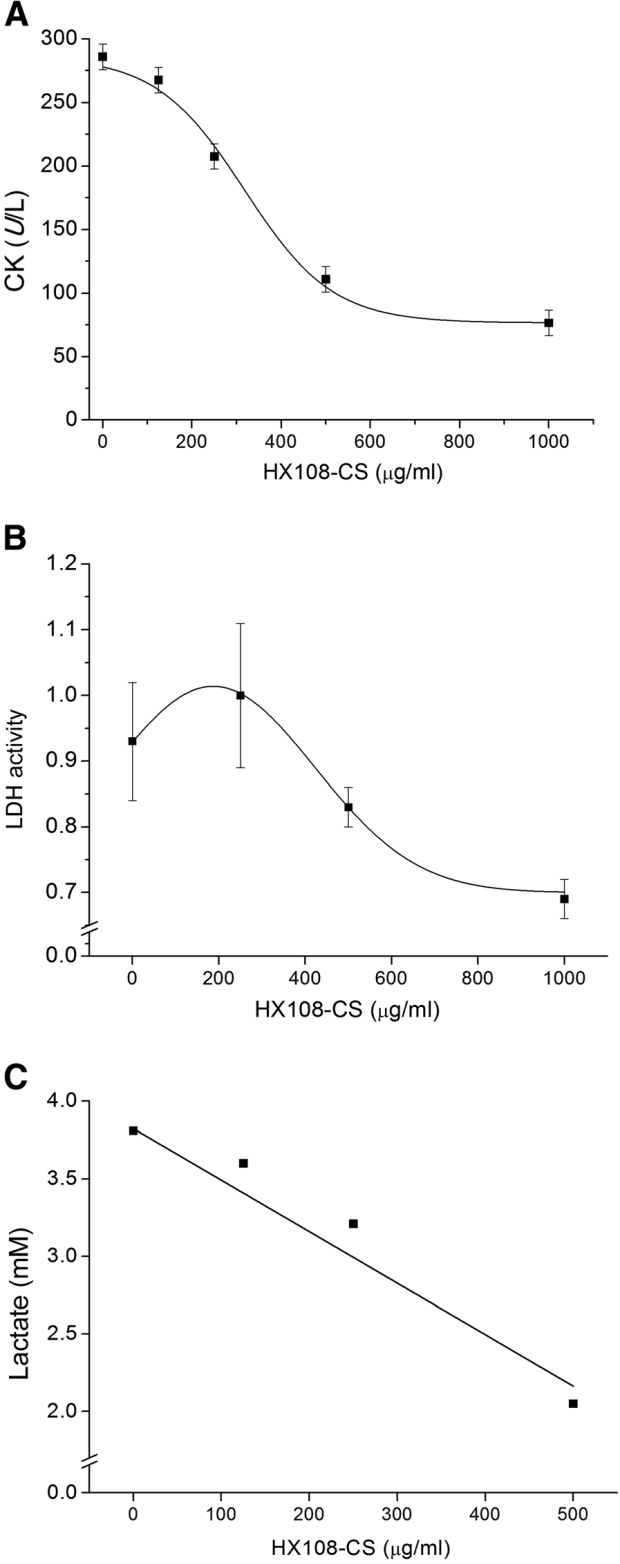
**Effect of HX108-CS on CK, LDH and lactate in C2C12 cells.** C2C12 cells were treated with HX108-CS (250, 500 and 1000 μg/ml) and 2, 4-dinitrophenyl (DNP) and sodium azide (NaN_3_) treatment for CK (**A**) and LDH activity (**B**), lactate (**C**) measurement respectively. Values are expressed as mean ± SEM.

HX108-CS was treated with graded concentration (0, 250, 500 and 1000 μg/ml) in C2C12 cells to examine the effect of HX108-CS on fatigue related parameters including lactate production, CK, and LDH activity. HX108-CS suppressed lactate production, CK, and LDH activity in a dose-dependent manner. As shown in Figure 3A, inhibition of CK activity was fit to the Hill equation, giving an EC_50_ of 323.23 ± 6.48 μg/ml. Also, when HX108-CS was treated with 500 μg/ml, HX108-CS induced inhibition of LDH activity was evaluated to 43.8 ± 2.5% of the control value (Figure 3B). Furthermore, HX108-CS suppressed the production of lactate by 50% in a dose of 250.23 ± 4.54 μg/ml (Figure 3C).

## Discussion

Major findings of current study were that i) oral HX108-CS (100 mg/kg) supplement significantly lowered the blood lactate accumulation immediately after high-intensity treadmill exercise and early recovery phase; ii) HX108-CS (100 mg/kg) treated mice improved the duration of swim-to- exhaustion that was concomitant with the decrease of lactate concentration; iii) HX108-CS inhibited lactate release, CK, and LDH activity in a dose-dependent manner (250, 500, and 1,000 μg/ml) *in vitro.* The present invention, named HX108-CS, relates to a composition which contains *Schisandra chinensis* and *Chaenomeles sinensis*. *Schisandra chinensis* extracts was reported as active ingredients for alleviating muscle fatigue [17], stress behavior [18], and enhancing exercise capability [10-14]. However, little is known about the effect of this new composition (HX108-CS) on lactate production, endurance performance, and the markers of muscle injury after high-intensity exercise.

We investigated to determine whether supplementation of HX108-CS suppresses exercise-induced elevation of lactate level, CK and LDH activities in rats (Figure 1). Our results showed that the blood lactate concentration in HX108-CS treated group was significantly lower than in the control group immediately after exhaustive exercise (Figure 1A) suggesting that lactate production was partially suppressed with HX108-CS supplementation compared with placebo group. Concurrently, removal of lactate during recovery period was significantly faster in HX108-CS treated group than placebo group.

On the contrary, CK and LDH activities were not significantly changed during and after exhaustive treadmill exercise and there was no statistical difference between HX108-CS and control groups. It may partly due to the dose of HX108-CS. The dose effective to elicit significant changes *in vitro* may not be high enough for changing in circulating levels. It would be worthwhile to try various high doses of HX108-CS for future study. In previous studies, serum CK activity has been shown to be elevated for 24 hours after exercise bouts, with a gradual return to basal levels in 72–96 hours [19,20], while serum LDH activity has been shown to be elevated 24 hours after exercise bouts and is maintained for 48–72 hours [21,22]. However, these previous studies were carried out in human subjects, and exercise intensity was also supposed to be higher than present study.

In addition, we conducted another *in vivo* experiment using mice to evaluate the effects of HX108-CS supplementation on endurance capacity using swimming exercise. We investigated whether or not oral supplementations of HX108-CS (0, 10, 50 and 100 mg/kg) would extend swimming time and suppress blood lactate concentration. Our results showed that duration of swimming time was prolonged significantly only in 100 mg/kg of HX108-CS group and swimming-induced lactate concentration was inhibited by both 50 and 100 mg/kg of HX108-CS groups (Figure 2). These results indicated that supplementation of HX108-CS is likely to be related to the improvement of endurance capacity, and the most effectual dose of HX108-CS in this regard was 100 mg/kg. To date, only one study reported different dose (30 mg/kg) of *Schisandra chinensis* had significant effect on the endurance capacity in mice with the measurement of blood lactate concentration [17]. In this study [17], the increase ratio of the blood lactate was also significantly lower in dose of 50 mg/kg of *Schisandra chinensis* aqueous extract consistent with the data of swimming endurance test. Possible explanations why doses were different between our study and previous study with respect to endurance capacity and lactate include (i) treatment period (14 days vs. 28 days), (ii) composition of supplement (mixed extract treatment vs. *Schisandra chinensis* only treatment) (iii) different species of mice (ICR vs. Kuming), (iv) exercise intensity (different weight bearing; 10% vs. 5% of body mass).

Finally, we investigated the effect of HX108-CS on lactate production, CK, and LDH activity *in vitro* using C2C12 cells (Figure 3). HX108-CS suppressed the CK, LDH activity and lactate production in a dose-dependent manner in C2C12 cells. Treatment of intact muscles with 2, 4-dinitrophenyl (DNP) has previously been shown to result in a decline in muscle ATP concentration [23] and increase of CK activity concentration [24]. Release of CK from skeletal muscle has long been used as an index of damage to muscle in both *in vitro* and *in vivo* studies. The CK reaction is of vital importance to skeletal muscle contraction. This reaction provides the most immediate means to replenish ATP in the cytosol. In this investigation, the DNP-induced increases of lactate production, CK, and LDH activities were inhibited by HX108-CS treatment (250, 500, and 1,000 μg/ml) in C2C12 cell. The results of *in vitro* study suggest that HX108-CS potentially exerts beneficial effects on muscle fatigue by alleviating muscle damage.

## Conclusions

In conclusion, our data suggest that the composition of the present invention (HX108-CS) may have beneficial effects on endurance capacity by lowering lactate accumulation. This may in part be related to an amelioration of skeletal muscle injury. Further studies to elucidate the underlying mechanisms how HX108-CS exerts anti-fatigue effect will be required. In addition, careful consideration of clinical trial with human participants will be a subject of a follow-up study.

## Abbreviations

LDH: Lactate dehydrogenase; CK: Creatine kinase; SSE: *Schisandra* seed extract; DW: Distilled water.

## Competing interests

The authors’ declare that they have no competing interest.

## Authors’ contributions

Oh, Chang, and Song were responsible for study design and interpretation of data. Oh and Kim (YA) drafted the manuscript and performed the statistical analysis. Kim (HJ) was mostly involved in acquisition of the data of this manuscript. Kim (DS), Ho, and Kim (SH) carried out in vitro experiments and provided detail methods sections. All authors read and approved the final manuscript.
